# Maternal exposure to gasoline and exhaust increases the risk of childhood leukaemia in offspring – a prospective study in the Norwegian Mother and Child Cohort Study

**DOI:** 10.1038/s41416-018-0295-3

**Published:** 2018-10-15

**Authors:** Jorunn Kirkeleit, Trond Riise, Tone Bjørge, David C. Christiani, Magne Bråtveit, Andrea Baccarelli, Stefano Mattioli, Bjørg Eli Hollund, Bjørn Tore Gjertsen

**Affiliations:** 1 Department of Occupational Medicine, Haukeland University Hospital, 5021 Bergen, Norway; 2 Department of Global Public Health and Primary Care, University of Bergen, Bergen, Norway; 3 Cancer Registry of Norway, Oslo, Norway; 4 Department of Environmental Health, Harvard TH Chan School of Public Health, Boston, USA; 5 Laboratory of Environmental Precision Biosciences, Mailman School of Public Health, Columbia University, New York, USA; 6 Department of Medical and Surgical Sciences, University of Bologna, Bologna, Italy; 7 Center for Cancer Biomarkers, Department of Clinical Science, Precision Oncology Research Group, University of Bergen, Bergen, Norway

**Keywords:** Cancer epidemiology, Cancer epidemiology

## Abstract

**Background:**

In the prospective population-based Norwegian Mother and Child Cohort Study (MoBa), comprising 113 754 offspring, we investigated the association between parental exposure to “gasoline or exhaust”, as a proxy for benzene exposure, and childhood leukaemia.

**Methods:**

Around gestational week 17, mothers and fathers responded to a questionnaire on exposure to various agents during the last 6 months and 6 months  pre-conception, respectively. Benzene exposure was assessed through self-reported exposure to “gasoline or exhaust”. Cases of childhood leukaemia (*n* = 70) were identified through linkage with the Cancer Registry of Norway. Risk was estimated by hazard ratios (HRs) with 95% confidence intervals (95%CI), comparing offspring from exposed and unexposed parents using a Cox regression model.

**Results:**

Maternal exposure to "gasoline or exhaust" was associated with an increased risk of childhood leukaemia (HR = 2.59; 95%CI: 1.03, 6.48) and acute lymphatic leukaemia (HR = 2.71; 95%CI: 0.97, 7.58). There was an increasing risk for higher exposure (*p* value for trend = 0.032 and 0.027). The association did not change after adjustment for maternal smoking.

**Conclusion:**

In spite of rather few cases, the findings in this prospective study, with the exposure metric defined *a priori*, support previous observations relating maternal exposure to benzene from gasoline and other petroleum-derived sources and the subsequent development of childhood leukaemia in the offspring.

## Introduction

Over the last two decades, childhood cancer incidence globally has increased by 13%.^1^ Of these, leukaemia is the most common, in Norway representing 30% of all cancer cases in 2012–2016 in the age group 0–14 years, with incidence rates per 100 000 person-years of 5.1 and 4.3 for males and females, respectively.^2^ Acute lymphocytic leukaemia (ALL) accounts for as much as 85% of all acute childhood leukaemia cases.^3^ However, the aetiology of childhood leukaemia remains largely unknown. Less than 5% of the cases are associated with known causes as inherited, predisposing genetic syndromes, or prenatal and childhood exposure to therapeutic ionising radiation^4^ and chemotherapy.^3^ Suggested risk factors include environmental exposure to benzene, air pollution, gasoline and other petroleum-derived products, pesticides, electromagnetic fields (above 3 µT), parental smoking, maternal coffee and alcohol consumption, advanced maternal age, infectious agents and population mixing, and maternal reproductive history.^3,5–10^

Benzene has been causally associated with adult leukaemia,^11^ and there is an accumulating number of studies suggesting benzene as a risk factor also for childhood leukemia.^12,13^ In studies assessing the association between benzene and childhood leukaemia, various proxies for benzene exposure have been used, including measured or modelled exposure to benzene or concomitant air pollution in outdoor air,^14–20^ residential proximity to heavy-traffic roads or petrol stations^15,17,21–23^ and through parental occupational exposure to products containing benzene.^24–28^ For the studies assessing parental occupational exposure to benzene, all but one recent census-based study^28^ are case-control studies and may be limited by a retrospective design with the inherent high risk of recall bias.

The present study is performed within the population-based Norwegian Mother and Child Cohort Study (MoBa) comprising 113 754 offspring (1999–2009), where we aimed to prospectively investigate whether parental self-reported exposure to “gasoline or exhaust”, used as a proxy for benzene exposure, reported prior to and/or during pregnancy was associated with a subsequent increased risk of childhood leukaemia in the offspring.

## Methods

### Study population and study design

The Norwegian Mother and Child Cohort Study (MoBa) is a prospective population-based cohort study conducted by the Norwegian Institute of Public Health.^29^ Participants were recruited from all over Norway from 1999 to 2008. During this period, pregnant women (index person) were recruited with a mailed invitation before a routine ultrasound examination, offered to all pregnant women in Norway at 17 weeks of gestation (www.fhi.no/morogbarn). The women consented to participation in 41% of the pregnancies. The cohort now includes 113 754 children, 94 428 mothers and 75 995 fathers. At 17 weeks, expecting mothers and fathers completed their first questionnaire comprising questions on demographic characteristics, occupational and environmental exposures, lifestyle factors and socioeconomic status. The current study was based on version 9 of the quality-assured data files released for research on benzene-induced childhood leukaemia in September 2016.

### Cancer ascertainment outcome

Incident cancer cases in the cohort of offspring were identified through linkage with the Cancer Registry of Norway,^30^ including all cancer cases reported up to December 31, 2014. The childhood cancers were categorised according to the International Classification of Childhood Cancer, version 3 (ICCC-3), which is based on ICD-O-3.^31^ The main outcome was childhood leukaemia (*n* = 70). Small numbers of cases precluded analysis of subtypes other than acute lymphocytic leukaemia (ALL, morphology codes 983539, 983639 and 983739, *n* = 52), representing 74% of the leukaemia cases. The cases’ first cancer diagnosis was used in the analysis.

### Assessment of environmental exposure

In the first questionnaire answered at pregnancy week 17th expectant mothers were asked about their work situation and leisure activities, including (question #68) “*Have you been in contact with any of the following substances either at work or in your leisure time during the last six months”*, thereafter listing a range of products, including several that comprise agents that have been suggested as leukaemogenic in the scientific literature (“gasoline or exhaust”, “oil-based paint”, “paint thinner, paint-, varnish/lacquer- or glue-remover or other solvents”,“motor oil, lubrication oil or other types of oil”, “formalin/formaldehyde” and “chemotherapy substances”). A similar question was asked to expectant fathers (question #20, “*Have you been exposed to any of the following in the six months before your partner became pregnant?”*), listing the same substances as in the questionnaire for expectant mothers. Hence, for the exposure to these substances, the exposure period reported for expectant mothers was 6 months prior to the 17th gestational week, and is a combination of exposure before conception and the first 17 weeks of pregnancy. For expectant fathers, the exposure period in question was six months prior to conception.

### Assessment of environmental exposure to gasoline and other petroleum-products containing benzene

Gasoline and petroleum-products are known sources of benzene exposure. Benzene is classified as a carcinogen and was according to Norwegian and European legislation in the relevant period of exposure (between 1999 and 2008 and thereafter), not allowed to be placed on the market, or used as a substance or as a constituent of mixtures in concentration greater than 0.1% by weight.^32^ An exception is for gasoline where a maximum content of benzene has been set to 1.0% by weight%.^32^

Since self-reported exposure to gasoline has the highest potential for exposure to benzene in the present study, the exposure to “gasoline or exhaust *(does not apply to filling your own car)"* was chosen as the main variable and used as a proxy of benzene-exposure. If the parent answered “yes” to being exposed, they were asked to write down the number of days of exposure during this 6-month period (daily = 180 days) prior to answering the questionnaire (pregnancy week 17). In order to differentiate parents with a higher exposure to gasoline or exhaust (e.g., occupational exposure, handling gasoline for hobbies, etc.) from parents having an exposure similar to the general population, we used a self-reported number of days ≥ 30 as a cut-off point decided upon* a priori*. This was based on an assumption that exposure below 30 days was less likely to be related to occupation, and therefore representing a lower exposure burden. The participants were then categorised as “never exposed”, “ever exposed” (1–180 days), “exposed 1–29 days” and “exposed 30–180 days”. “Never-exposed” were chosen as the reference category in all analyses. Only the leukaemia cases with information on the respective exposures are included in the analysis.

Excess risk of childhood leukaemia after parental exposure to petroleum-derived products (solvents, aliphatic and aromatic hydrocarbons, mineral oils, paint, etc.) has been reported.^12,27,33^ Hence, in order to assess the risk of exposure to other aromatic and aliphatic hydrocarbons, self-reported exposure to “oil-based paint” and “paint thinner, paint-, varnish/lacquer- or glue-remover or other solvents” obtained from the same questionnaires, were included. However, due to regulations on benzene,^32^ these products were not likely to contain benzene beyond trace amounts («0.1 weight%) in the relevant time period.

### Assessment of exposure to other leukemogenic agents

The known leukaemogenic agents “formalin/formaldehyde”^34^ and “chemotherapy substances (*not including your own medical treatment*)”^35,36^ were also included. Exposure to these agents were also categorised in “no exposure” (0 days), 1–29 days or 30–180 days.

Information on ionising radiation was divided into paternal exposure preconception (“worked with X-ray equipment” or “had any X-rays taken”) and maternal exposure during pregnancy (“worked with X-ray equipment”). We did not have information on offspring’s exposure to ionising radiation *in utero*or in early childhood.

### Other risk factors and potential confounders

High birth weight,^37,38^ low maternal intake of folate,^39,40^ parental^41,42^ and maternal grandmother’s smoking,^43^ advanced maternal age^44^, maternal consumption of alcohol,^8,9^ as well as high socioeconomic status^45^, have all been suggested to increase or mediate the risk of childhood leukaemia. Information on offspring’s birthweight (continuous), maternal intake of folic acid supplements before and during pregnancy (no/yes/unknown), maternal smoking during pregnancy (no/yes/unknown), paternal smoking 6 months prior to conception (no/yes/unknown), maternal grandmother’s smoking (no/yes/unknown), maternal (continuous and categorical) and paternal (categorical) age, parental consumption of alcohol (categorical), proxies of socioeconomic status, including maternal education (six categories from public school to > 4 years at university/college) and maternal marital status (married/cohabitant or other, categorical), were obtained from the same self-administered questionnaire as the main exposure variable or from the Medical Birth Registry of Norway (Table 1). Documentation on the questionnaires administered on pregnancy week 17 is available at the cohort study’s website.^46^

**Table 1:** Characteristics of the study population (offspring) and parents (mothers and fathers), the Norwegian Mother and Child Cohort Study, Norway, 1999–2009

Variable		Offspring (all)	Cases of leukemia
Number of subjects		113 754	70
Gender^a^	Male (%) Female (%)	58 200 (51.2) 55 330 (48.6)	29 (41.4) 41 (58.6)
Gestational age (weeks)^a^	Mean (SD)	39.4 (2.0)	39.0 (2.1)
Birth weight (kg)^a^	Mean (SD)	3 561 (599)	3 614 (745)
Maternal age at birth (years)^a^	Mean (SD)	30.1 (4.7)	30.0 (4.8)
Maternal intake of folic acid supplemen tation^a^ *Before* *pregnancy*	No (%) Yes (%)	83 703 (73.8) 29 758 (26.2)	53 (75.7) 17 (24.3)
*During* *pregnancy*	No (%) Yes (%)	49 032 (43.2) 64 429 (56.8)	28 (40) 42 (60)
Variable		Mother (n = 94 428)	Father (n = 75 995)
Parental age in categories (%)^a^	<20 years (%) 20–29 years (%) 30–39 years (%) ≥40 years (%) 40–49 years (%) ≥50 years (%)	1340 (1.2),48 811 (42.9) 61 032 (53.7) 2 348 (2.1)	409 (0.4) 30 967 (27.4) 70 277 (61.8) 12 101 (10.6) 10 722 (9.4) 812 (0.7)
Parental level of education^b^	Level 1 (%) Level 2 (%) Level 3 (%) Level 4 (%) Level 5 (%) Level 6 (%) Missing (%)	2 804 (2.5) 4 941 (4.3) 12 548 (11.0) 14 236 (12.5) 39 181 (34.4) 22 428 (19.7) 17 616 (15.5)	4 624 (4.16) 5 570 (4.9) 23 850 (21.0) 11 441 (10.1) 24 985 (22.0) 21662 (19.0) 21 622 (19.0)
Parental smoking	No (%) Sometimes/daily (%)Don’t know/missing (%)	82 788 (90.6) 8497 (9.3) 22469 (19.8)	22 527 (49.1) 23 361 (50.9) 67 866 (59.6)
Maternal grandmother smoking	No (%) Yes (%) Don’t know/missing (%)	65 793 (57.8) 2 506 (22.0) 22 894 (20.1)	NA
Parental alcohol consumption^c^	Never (%) <1 per week (%) 1 per week (%) >1 per week (%) Missing (%)	75 710 (87.1) 10 699 (12.3) 428 (0.49) 83 (0.10) 26 834 (23.6)	1 721 (2.3) 41 038 (54.5) 18 594 (24.7) 13 972 (18.5) 38 429 (33.8)

*SD* standard deviation, *NA* not applicable

^a^Source MFR

^b^Level of education: 1 = 9 year secondary school, 2 = 1–2 year high school, 3 = 3-year high school general studies, junior college, 4 = University/college 4 years (Bachelor’s degree or similar), 5 > 4 years (master’s degree, medical doctor, PhD or similar)

^c^Maternal consumption = during pregnancy, paternal consumption = pre-conception

### Statistical analysis

We estimated the risk of developing childhood cancer using Cox proportional hazard regression models giving hazard ratios (HRs) with 95% confidence intervals (95% CI) comparing the exposed with the unexposed children. An early onset of the childhood cancer does not necessarily indicate a stronger association to exposure. Therefore, we did not model the risk according to age at onset of disease, and used a constant (time = 1) for indicating time to disease for the cases or time to end of study for those who did not develop cancer. We used the “cluster” option in the STCOX command in STATA to account for intragroup correlation between siblings within the dataset.

We tested potential suggested confounders, including birthweight, maternal intake of folate, parental and maternal grandmother’s smoking, parental age, parental consumption of alcohol, and socioeconomic status (level of education and marital status), by estimating the effect of these factors on the outcome. To test for confounding by maternal smoking, we also estimated the association between exposure to benzene and risk of childhood leukaemia among non-smokers only. A test for trend across the three levels of exposure was performed by including the categorical variable as a continuous variable in the model.

We tested for effect modification by parental age and offspring sex by introducing an interaction term in the model. Associations with *p* < 0.05 were considered statistically significant. The data were analysed using the software package STATA IC/15.0 (StataCorp, College Station, TX, USA).

## Results

The final cohort included 113 754 children (51.2% males and 48.6% females), 94 428 expecting mothers and 75 995 expecting fathers (Table 1). Average follow-up time for the cohort and age at diagnosis of leukaemia were 9.4 years (SD 2.2, range 0–15) and 3.7 years (SD 2.7, range 0–11), respectively. By the end of follow-up 199 children were diagnosed with childhood cancer (all sites), of which 70 cases (35.2%) were leukaemia (ALL; *n* = 52 (74.3%), acute myelogenic leukaemia (AML); *n* = 16 (22.9%)). Females were overrepresented among the leukaemia cases (58.6%) (Table 1). No statistically significant differences between the leukaemia cases and the other offspring in the cohort were found for sex, gestational week, birth weight, maternal intake of folic acid supplementation, parental age, parental smoking, parental consumption of alcohol, education or marital status (*Χ*^2^ tests and *t* tests, all *p* values > 0.05).

We found no increased risk of overall childhood cancer (all sites) among offspring having parents reporting exposure to “gasoline or exhaust” (Table 2). However, for childhood leukaemia there was an increased risk (HR: 2.59; 95% CI: 1.03, 6.48) for maternal self-reported exposure to “gasoline or exhaust”. The risk increased with number of days being exposed during the last six months prior to pregnancy week 17 (*p* value for trend = 0.032). Including only ALL-cases, the risk estimates gave a slightly stronger estimate of 4.03 (95% CI: 1.25, 13.0) in the highest exposed group with a *p* value for trend of 0.027 (Table 2). The risk for ALL was in excess also for paternal exposure to “gasoline and exhaust” during the 6 months prior to conception, but only statistically significant in the lowest exposed group (HR: 2.60; 95% CI: 1.07, 6.32). The number of AML-cases (*n* = 16) did not allow us to perform separate analysis for this histological subtype. No excess risk of leukaemia (all combined) was found for paternal exposure.

**Table 2:** Unadjusted hazard ratios (HRs) for the effect of parental exposure to “gasoline or exhaust” (excluding filling their own car) on offspring’s risk of childhood cancer (overall) and leukemia, the Norwegian Mother and Child Cohort Study, Norway, 1999–2009

Neoplasm	Exposure metric	Maternal exposure^a^				Paternal exposure^a^			
		*N* (94 428)	Cases (n)^b^	HR	95% CI	*N* (75 995)	Cases (n)^b^	HR	95% CI
All cancer	Non exposed	90 984	154	1.00	Referent	65,231	103	1.00	Referent
	Ever	3 444	7	1.20	0.56, 2.56	10,761	21	1.25	0.95, 1.65
	1–29 days	1 706	3	1.04	0.33, 3.25	5780	13	1.42	0.80, 2.53
	30–180 days	1 738	4	1.36	0.50, 3.66	4981	8	1.02	0.50, 2.09
Leukemia (all subtypes)	Non exposed	90 984	51	1.00	Referent	65,234	35	1.00	Referent
	Ever	3 444	5	**2.59**	**1.03, 6.48**	10,761	8	1.34	0.84, 2.15
	1–29 days	1 706	2	2.09	0.51, 8.58	5780	6	1.93	0.81, 4.60
	30–180 days	1 738	3	3.08	0.96, 9.86	4981	2	0.75	0.18, 3.11
	*p* value for trend	*p* *=* 0.032				*p*= 0.74			
Acute lymphatic leukemia (ALL)	Non exposed	90 984	39	1.00	Referent	65,231	26	1.00.	Referent
	Ever	3 444	4	2.71	0.97, 7.58	10,761	8	1.34	0.78, 2.31
	1–29 days	1 706	1	1.37	0.19, 9.94	5780	6	**2.60**	**1.07, 6.32**
	30–180 days	1 738	3	**4.03**	**1.25, 13.0**	4981	2	1.01	0.24, 4.24
	*p* value for trend	*p* = 0.027				p = 0.27			

^a^For mothers the period of self-reported exposure refers to the last 6 months prior to 17th week of pregnancy, while it for fathers refers to 6 months prior to conception. ^b^Only the leukemia cases with information on the respective exposures are included in the analysis. Statistically significant associations are in bold font.

We found no associations between any of the potential confounders and the risk of leukaemia. When excluding mothers smoking at pregnancy week 17 or unknown smoking status, the risk estimates did not change substantially (leukaemia HR: 2.71; 95% CI: 0.97, 7.55). Further, we found no effect modification by offspring sex (p for test of interaction = 0.30), maternal age below versus above 30 years of age (*p* = 0.79) or paternal age below versus above 40 years of age (*p* = 0.70).

Table 3 shows the HRs of childhood cancer (all types) and leukemia related to exposure to other chemicals suggested to be risk factors for childhood leukemia. Self-reported parental exposure to “organic solvents” or “oil-based paint” did not increase the risk of childhood (overall) cancer or leukemia. However, maternal exposure to formaldehyde and antineoplastic agents used in chemotherapy (not including as own medical treatment), both known leukaemogenic agents, gave a 13-fold increased risk of leukaemia in the offspring. The excess risk for both these exposures was ascribed to two cases of leukaemia being born to mothers reporting an exposure to formaldehyde (*p* value for trend = 0.067), and two other mothers being exposed to antineoplastic drugs (*p* value for trend = 0.021) of ≥30 days during the last six months prior to pregnancy week 17. No excess risk of leukaemia was found for paternal exposure to these agents.

**Table 3:** Unadjusted hazard ratios (HRs) for the effect of parental exposure to chemical agents on offspring’s risk of childhood leukemia (total, all subtypes), the Norwegian Mother and Child Cohort Study, Norway, 1999–2009

Agent	Exposure metric	Maternal exposure^b^				Paternal exposure^b^			
		*N*	Cases (n)^c^	HR	95% CI	*N*	Cases (n)^c^	HR	95% CI
Organic solvents^a^	Non exposed	72 275	39	1.00	Referent	37 736	22	1.00	Referent
	Ever	21 157	15	1.31	0.72, 2.38	38 209	21	0.94	0.52, 1.71
	1–29 days	20 141	14	1.29	0.70, 2.37	34 577	21	1.04	0.57, 1.89
	30–180 days	1 016	1	1.83	0.25, 13.2	3 632	0	—	—
	*p* value for trend	*p* = 0.34				*p*= 0.43			
Oil-based paint	Non exposed	70 524	37	1.00	Referent	42 652	25	1.00	Referent
	Ever	25 168	18	1.36	0.78, 2.39	33 720	18	0.91	0.50, 1.67
	1–29 days	24 028	17	1.35	0.76, 2.39	31 367	17	0.92	0.50, 1.71
	30–180 days	1 140	1	1.67	0.23, 12.2	2 353	1	0.73	0.10, 5.35
	*p* value for trend	*p* = 0.27				*p* = 0.72			
Formaldehyde	Non exposed	91 694	52	1.00	Referent	74 499	43	1.00	Referent
	Ever	1 826	2	1.93	0.47, 7.92	1 116	0	—	—
	1–29 days	1 560	0	—	—	962	0	—	—
	30–180 days	266	2	**13.26**	**3.24, 54.2**	154	0	—	—
	*p* value for trend	*p* = 0.067							
Chemotherapy substances (not own treatment)	Non exposed	92 211	52	1.00	Referent	75 183	43	1.00	Referent
	Ever	1 278	2	2.78	0.68, 11.42	416	0	—	—
	1–29 days	1 007	0	−	—	345	0	—	—
	30–180 days	271	2	**13.2**	**3.19, 54.4**	71	0	—	—
	*p* value for trend	*p* = **0.021**							

^a^Paint-thinner, paint-, varnish/laquer- or glue-remover or other solvents (e.g., Lynol^®^, white spirit, toluene, carbon tetrachloride). ^b^For mothers the period of self-reported exposure refers to the last 6 months prior to 17th week of pregnancy, while it for fathers refers to 6 months prior to conception. ^c^Only leukemia cases with information on the respective exposures are included in the analysis. Statistically significant associations in bold font

## Discussion

We found that children born to mothers who reported exposure to “gasoline or exhaust”, a known source of benzene exposure, during the six months prior to pregnancy week 17 had a subsequent excess risk of leukemia (overall) and ALL. The risk increased with number of days these mothers had been exposed, and could not be explained by any of the other suggested risk factors assessed in the present study. Hence, this cohort study supports results from a recent census-based cohort study,^28^ and the increasing number of retrospective case-control studies and meta-analyses, suggesting maternal benzene exposure as a risk factor for childhood leukemia.^12,33^

We did not find any increased risk of childhood leukemia for paternal self-reported exposure to “gasoline or exhaust”. A weaker association between paternal exposure and childhood leukemia is in line with the findings in other studies on paternal benzene exposure, including the census-based cohort study where only maternal occupational exposure to benzene was associated with childhood leukemia (HR 1.73; 95% CI: 1.12–2.67) and ALL (HR: 1.88; 95% CI: 1.16–3.04).^28^ A meta-analysis of cancer-incidence studies assessing the effect of benzene exposure after handling occupational and household products found a summary relative risk (sRR) for childhood leukemia of 1.96 (95% CI: 1.39, 2.78) for maternal exposure compared to sRR = 1.23 (95% CI: 1.07, 1.41) for paternal exposure.^12^ When only including the metric occupational exposure to benzene, the corresponding sRRs were 1.71 (95% CI: 1.14, 2.58) for maternal exposure and 1.18 (95% CI: 1.00, 1.41) for paternal exposure. In the SETIL-study that included parental exposure to “petrol exhaust” and “diesel exhaust”, comparable to the exposure metric used in our study, the risk estimates were higher for mothers’ exposure, but statistically significant only for the fathers’ exposure likely due to a higher frequency of exposure.^27^

The foetus is vulnerable to environmental exposure that disrupts developmental processes during relatively narrow time windows.^47^ Given that benzene passes the foetal blood stream through the maternal-foetal unit also in humans,^48^ and that the haematopoiesis starts in the liver and thymus from the seventh and ninth week post conception,^49^ respectively, the first trimester might be an important susceptibility window with respect to later development of environmental-induced hematopoietic malignancy. The information on timing of the exposure in the present study did not allow us to differentiate between the exposure prior to conception and during the first 17 weeks of pregnancy (*in utero*). Of case-control studies differentiating between time windows for maternal exposure, Miligi et al.^27^ reported an excess risk of leukaemia across all time windows for maternal exposure to aromatic hydrocarbons. However, it was only statistically significant for preconception (odds ratio (OR) 3.8, 95% CI: 1.6–9.2) and during pregnancy (OR 2.2, 95% CI: 1.0–9.2) for leukaemia (all), and for preconception for ALL (OR: 3.8, 95% CI: 1.5–9.5). The same trend was reported for diesel exhaust and gasoline exhaust, but the ORs were not statistically significant. For studies using air pollution as a proxy of benzene exposure, Heck et al.^18^ reported an excess risk of ALL (OR: 1.50, 95% CI: 1.08–2.09) and AML (OR: 1.75, 95% CI: 1.04–2.93) for benzene exposure in ambient air during 3rd trimester, but not for 1st and 2nd trimester. Hence, present knowledge does not allow us to differentiate risk of childhood leukaemia according to exposure susceptibility time windows.

The low numbers of exposed parents is a general limitation of cohort studies on benzene in the general population. In the present study, the prevalence of exposure to “gasoline or exhaust” among mothers was low, being 3.6% and 1.8% for “ever exposed” and “ ≥ 30 days exposed”, respectively, for the last 180 days. The corresponding prevalence for fathers were 14.2% and 6.6%. These numbers are in accordance with the Swiss census-based cohort study, where 5.8% of the mothers and 14% of the fathers were classified as occupationally exposed to benzene using information on reported occupations and a job exposure matrix (JEM).^28^ Among the controls in a case-control study, 1.6% of the mothers and 17% of the fathers reported being occupationally exposed to diesel or petrol exhaust during pregnancy and pre-conception, respectively.^27^

The information on exposure in our study was based on self-report, as also was the case for the previous case-control studies using job histories or indicated specific exposures. Self-reported and parent-reported exposures are prone to misclassification. In retrospective studies, this misclassification might often be differential by disease status and thereby introducing bias.^50,51^ In the present study, all exposure reports were done prior to knowledge of any outcome, excluding differential misclassification caused by i.e., differential recall. Further, the likely non-differential exposure misclassification present in our study would in general bias the results towards the null. Finally, the cut-off point for categorising number of days (30 during the last 180 days) being regularly exposed and used for the dose–response analysis was decided *a priori*, and not influenced by the data.

All studies assessing the association between benzene exposure and risk of childhood leukaemia, including the present one, have used an indirect measure of benzene exposure, including air pollution, use of petroleum-based products or residential proximity to benzene sources. In the meta-analysis by Carlos-Wallace and coworkers^12^ it was reported that several metrics and common sources of benzene exposure were associated with childhood leukaemia. For parental and early childhood exposure, including both occupational and household product use, the risk was increased for leukaemia combined (sRR: 1.96; 95% CI: 1.6, 2.4), ALL (sRR: 1.57; 95% CI: 1.3, 1.9) and AML (sRR: 2.34; 95% CI: 1.7, 3.2). When including studies using traffic-related air pollution as the surrogate of benzene, sRRs of 1.49 (95% CI: 1.2, 2.5) for ALL and 2.07 (95% CI: 1.3, 3.2) for AML were found. Combining the subtypes of leukaemia and only including studies using residential proximity to gasoline stations as the proxy for benzene exposure, the summary relative risk for leukaemia increased to 2.42 (95% CI: 1.51,3.89).^52,53^ This meta-analysis includes studies performed in both the developed and developing world published between 1987 and 2014, and it is hence difficult to say something general about the level of benzene in the products and exposure scenarios assessed. However, although gasoline, paint and degreasers historically have been a significant source of benzene exposure, benzene is at least in the developed world today found in gasoline below 1 weight% and only as a contamination («0.1%) in petroleum-based solvents and paints. Hence, overall these observations, together with the risk estimates reported in the present study, indicate that relatively low exposure to benzene might be associated with an increased risk of childhood leukaemia.

Further, “gasoline or exhaust” is a complex mixture of many compounds other than benzene, including carcinogenic agents such as polycyclic aromatic hydrocarbons (PAHs), 1,3-butadien and various nitroarenes, aldehydes and heavy metals.^54^ We cannot exclude possible contribution of other co-exposures, but there is little evidence that these compounds increases the risk of adult or childhood leukemia at the relevant concentrations.^54,55^ Clearly, there is a need for prospective or nested case-control studies with more detailed and accurate measures of benzene exposure that could evaluate the relation to the risk of childhood leukemia in a dose–response manner. However, such studies face challenges related to statistical power.

Passive and active exposure to cigarette smoke is a known source of benzene and other carcinogenic agents. In addition to benzene emitted into ambient air during refuelling of petrol and incomplete combustion of petrol and other organic materials, active and passive smoking of cigarettes are the main sources of benzene exposure in the general population. Cigarette smoking has also been associated with adult AML, while the results for childhood leukaemia have been inconclusive.^41^ The evidence for an association has been strongest for the AML-subtype also for children. However, in a recent pooled analysis of the Childhood Leukemia International Consortium (CLIC), and a meta-analysis of CLIC and non-CLIC studies, no association with maternal smoking before, during or after pregnancy was found for childhood AML.^42^ In the present study, the association between maternal exposure to “gasoline or exhaust” and childhood leukaemia risk did not change after adjustment for maternal smoking or after excluding mothers who smoked during pregnancy from the analysis. The validity of self-reported tobacco use as a marker of tobacco exposure has been shown to be high in this cohort evaluated by correlating urinary cotinine levels to self-reported tobacco use.^56^

High birthweight has consistently been reported to be associated with an increased risk of childhood leukemia.^37,38,57,58^ The International Childhood Cancer Cohort Consortium (I4C), including the present MoBa-cohort, also reported that childhood cancer incidence, including leukaemia, rises with increasing birthweight.^38^ In the present study, we found no effect of birth weight on the risk for childhood cancer, and the risk estimates related to reported paternal and maternal exposure to “gasoline or exhaust” did not change after including birth weight in the model.

Results from studies assessing the effect of maternal intake of folic acid on the risk of childhood leukaemia have been mixed, with evidence for a protective effect in the majority of studies.^39,40,59^ In a recent study on Norwegian offspring followed in the same period as in MoBa, no association between measures of maternal intake of folate and leukaemia was found.^60^ The measures of dietary supplementation of folic acid did not affect the association between exposure to “gasoline or exhaust” and leukaemia reported in the present study.

In addition to ascertaining exposure to benzene, the present study also included information on a few other leukaemogenic agents. We found a statistically significant 13-fold increased risk of childhood leukaemia associated with maternal exposure to antineoplastic drugs and formaldehyde, both classified as leukaemogenic agents.^34–36^ No increased risk was found for the other exposures examined. Our findings on antineoplastic drugs and formaldehyde were based on only two cases for each exposure and should be interpreted with caution. Nevertheless, the risk estimates were worrisome, and an enhanced focus on exposure reduction measures among health personnel handling these agents is probably needed.

The response rate of the Norwegian Mother and Child Study study was 41%, having a higher percentage of participants with a socioeconomic status above the population mean and an underrepresentation of the youngest women (<25 years), those living alone and smokers.^29,61^ However, a study of self-selection and bias in the present cohort suggested that estimates of exposure-outcome associations were not biased.^61^ The measures of socioeconomic status in the presents study, including level of maternal education, did not affect the association between exposure to “gasoline or exhaust” and leukaemia.

In conclusion, in a prospective study where exposure to “gasoline or exhaust” was reported among ~94 000 mothers and 75 000 fathers and where 113 000 children were followed for development of childhood leukemia, we found an excess risk increasing with number of days being exposed. Due to the small number of cases reflected in the wide confidence intervals, and a lack of detailed data on benzene exposure, the results must be interpreted with caution. Nevertheless, these findings in a prospective study with definition of the exposure metric a priori supports the previously identified relationship between maternal exposure to petroleum-derived benzene, including gasoline, and subsequent development of childhood leukemia in the offspring.
